# Geographic Atrophy in Patients with Age-Related Macular Degeneration Is Associated with Rare Variants in Complement Factor H and Complement Factor I

**DOI:** 10.1016/j.xops.2026.101171

**Published:** 2026-03-27

**Authors:** Anita de Breuk, Sarah de Jong, Bjorn Bakker, Eveline Kersten, Daniel T. Luttikhuizen, Sascha Fauser, Caroline C.W. Klaver, Anneke I. den Hollander, Carel B. Hoyng, Yara T.E. Lechanteur

**Affiliations:** 1 Department of Ophthalmology, Radboud University Medical Center, Nijmegen, The Netherlands; 2 Department of Ophthalmology, Rijnstate, Arnhem, The Netherlands; 3 Department of Ophthalmology, Erasmus Medical Center, Rotterdam, The Netherlands; 4 Department of Epidemiology, Erasmus Medical Center, Rotterdam, The Netherlands; 5 Department of Ophthalmology, University Hospital of Cologne, Cologne, Germany; 6 Roche Pharma Research and Early Development, F. Hoffmann-La Roche, Basel, Switzerland; 7 Institute of Molecular and Clinical Ophthalmology, Basel, Switzerland; 8 Ophthalmology Research, Sanofi, Cambridge, Massachusetts

**Keywords:** Age-related macular degeneration, Complement factor H, Complement factor I, Geographic atrophy, Phenotype

## Abstract

**Purpose:**

To describe the phenotype of patients with age-related macular degeneration (AMD) carrying rare genetic variants in the complement factor H (*CFH*) and complement factor I (*CFI*) genes.

**Design:**

Cross-sectional study.

**Participants:**

Two hundred thirty-four patients with AMD carrying rare variants in *CFH* (n = 134) and *CFI* (n = 100) and 234 AMD noncarriers.

**Methods:**

Genetic data of patients with AMD from the European Genetic Database were filtered for rare coding and splice-site variants in *CFH* and *CFI*. For each carrier, an age-matched (±2 years) patient with AMD without rare variants in *CFH* and *CFI* (noncarrier) was selected. Phenotypic characteristics on color fundus photographs were graded according to the Rotterdam Classification and compared between carriers and noncarriers by univariate generalized estimating equations with binary logistic regression analyses, applying a Bonferroni correction for multiple comparisons. We performed subanalyses for pathogenic rare variants only, and we analyzed *CFH* and *CFI* carriers separately.

**Main Outcome Measures:**

Phenotypic characteristics on color fundus photographs.

**Results:**

Geographic atrophy and intermediate AMD, along with features such as predominant drusen type, largest drusen size, and drusen area, were associated with carriership of rare pathogenic variants in *CFH* (*P* < 0.001, *P* = 0.002, *P* < 0.001, and *P* < 0.001, respectively). Geographic atrophy and intermediate AMD, along with features such as drusen size, drusen area, and pigmentation, were associated with carriership of rare pathogenic variants in *CFI* (*P* = 0.01, *P* = 0.006, *P* < 0.001, and *P* = 0.006, respectively). Furthermore, carriers of rare pathogenic variants in *CFH* were younger (*P* < 0.001) and had a lower genetic risk score for common AMD-associated variants compared with noncarriers (mean [standard deviation] genetic risk score 0.83 [1.01] vs. 1.41 [1.21], *P* = 0.03).

**Conclusions:**

In this study, patients with AMD carrying rare variants in *CFH* and *CFI* had a more severe drusen phenotype, and a higher frequency of geographic atrophy at a relatively early age. Identifying this distinct phenotype could aid in pinpointing individuals who are more likely to benefit from complement-inhibiting therapies.

**Financial Disclosure(s):**

The authors have no proprietary or commercial interest in any materials discussed in this article.

Age-related macular degeneration (AMD) is one of the leading causes of severe visual impairment in the elderly.^1^ The disease is characterized by drusen and pigmentary changes in the early stages. In the course of the disease, geographic atrophy (GA) or choroidal neovascularization (CNV) can develop, leading to severe visual impairment. Both genetic and nongenetic factors, such as age and smoking, are involved in the disease etiology.^2^, ^3^, ^4^ With respect to genetic factors, 52 variants in different disease pathways are reported to be independently associated with AMD.^5^ Most of these variants (45/52) are common risk-increasing or risk-decreasing variants with, in general, moderate effect sizes. The other 7 variants are rare and, in several cases, have larger effect sizes. These rare variants are predominantly located in genes of the complement pathway (e.g., complement factor H [*CFH*], complement factor I [*CFI*], complement component 3 [*C3*], and complement component 9 [*C9*]), pointing toward an important role of this pathway in AMD pathogenesis.^5^, ^6^, ^7^, ^8^, ^9^, ^10^ To date, a large number of rare variants in complement genes have been identified in AMD case-control and family studies;^11^^,^^12^ however, the functional consequences of these variants are largely unknown. Multiple clinical trials studied the effect of complement inhibition in patients with AMD. Although most were unsuccessful, several recent clinical trials demonstrated a reduction in GA growth in patients treated with complement inhibitors, leading to 2 Food and Drug Administration-approved drugs (C3 inhibitor pegcetacoplan, SYFOVRE; and C5 inhibitor avacincaptad pegol, IZERVAY) for the treatment of GA secondary to AMD.^13^^,^^14^ One might argue that patients with AMD conferring the highest genetic risk in the complement pathway (for example, patients who carry rare variants in *CFH* or *CFI* that result in reduced factor H or factor I levels or in impaired factor H or factor I function) benefit most from therapies targeting the complement pathway. Two clinical trials applied such a strategy, and selected patients based on *CFH* and *CFI* genotype (GEM103, NCT04246866, intravitreal supplementation of factor H; GT005, NCT04437368, subretinal *CFI* gene therapy); however, these trials were terminated early, and reasons for termination are not always clearly reported. Nevertheless, it remains an important question whether patients with AMD carrying rare variants in complement genes respond better to complement inhibition. Studying the phenotype of patients carrying rare variants in complement genes might help to better recognize these carriers. For example, ophthalmologists could examine the retina of patients with AMD for the presence of specific features that support the suspicion of carriership of rare variants in complement genes. In 2017, Kersten et al^15^ studied color fundus photographs (CFP) of 51 patients with AMD carrying rare variants in *CFH* and found that carriers of pathogenic rare variants in *CFH* present more often with an extensive drusen area in the macula, drusen nasal to the optic disc, and crystalline drusen, compared with patients with AMD without rare variants in *CFH*.^15^ Recently, we have expanded our cohort of carriers of rare variants in *CFH*^15^ and also included a cohort of patients with rare variants in *CFI*. In this study, we aimed to evaluate phenotypic features in patients with AMD carrying rare variants in *CFH* and *CFI*, and to compare these characteristics to those of patients with AMD without rare variants.

## Methods

### Study Population

All patients included in this study were recruited from the European Genetic Database (EUGENDA), a large multicenter database for clinical and molecular analysis in AMD, consisting of patients with AMD and control individuals from the Radboud University Medical Center (Nijmegen, The Netherlands) and from the University Hospital of Cologne (Cologne, Germany). Patient recruitment took place from June 2004 to December 2020. This study was approved by the local ethical committees of the Radboud University Medical Center and the University Hospital of Cologne and adhered to the tenets of the Declaration of Helsinki. All study participants provided written informed consent.

For this study, we selected patients with AMD who carried a rare variant in *CFH* or *CFI*, and patients with AMD without rare variants in *CFH* or *CFI*, hereafter referred to as “noncarriers.” Carriers were defined as patients with AMD carrying at least 1 rare genetic protein-altering or splice-site variant in either the *CFH* or *CFI* genes (minor allele frequency < 1% in non-Finnish Europeans as reported in the Genome Aggregation Database, http://gnomad.broadinstitue.org). Noncarriers were defined as patients with AMD who did not carry any rare variants in *CFH*, *CFI* or other complement genes (*C3*, *C9*, and complement factor B). For each carrier, we selected an age-matched (±2 years) noncarrier at a 1:1 ratio. In total, 139 carriers of rare variants in *CFH*, 101 carriers of rare variants in *CFI*, and 1 carrier of a rare variant in both *CFH* and *CFI* were identified. Because this study is a continuation of the work performed by Kersten et al,^15^ it is important to note that 45 carriers of rare variants in *CFH* overlapped with the previous study.

### Image Acquisition and Grading

CFPs centered on the fovea were obtained using a Topcon TRC 50IX fundus camera (Topcon), Canon UVI fundus camera (Canon), or Topcon DRI Triton camera (Topcon), and optical coherence tomography (OCT) images were captured by using Spectralis HRA+OCT (Heidelberg Engineering). Features of AMD were graded on CFPs of the carriers and noncarriers by experienced graders of the EyeNED Reader Center, according to the Wisconsin Age-Related Maculopathy Grading System^16^^,^^17^ and stratified into stages according to the Rotterdam Classification.^18^ Phenotypic features included predominant drusen type, crystalline drusen, extramacular drusen, drusen nasal of the optic disc, largest drusen size, area (%) of the early treatment diabetic retinopathy study (ETDRS) grid covered by drusen, increased pigment, retinal pigment epithelium (RPE) degeneration, GA, and CNV.

### Genotyping

Individuals from the EUGENDA database underwent extensive genotyping according to one or more of the following procedures: whole-exome sequencing,^19^ single-molecule molecular inversion probes in combination with next-generation sequencing,^20^ or exome chip analysis (custom-modified HumanCoreExome array),^5^ which was available for >4000 individuals in EUGENDA. In addition, competitive, allele-specific, polymerase chain reaction assays (Kompetitive Allele Specific PCR Single Nucleotide Polymorphisms Genotyping; LGC Group) for the rare variants *CFI* c.392T>G (p.Leu131Arg), *CFI* c.1657C>T (p.Pro553Ser), *CFH* c.524G>A (p.Arg175Gln), *CFH* c.578C>T (p.Ser193Leu), and *CFH* c.3628C>T (p.Arg1210Cys) and a custom-made assay (TaqMan; Life Technologies) for the rare variant *CFI* c.355G>A (p.Gly119Arg) were previously performed in a subset of the EUGENDA database. In a small subset, a panel of complement genes was sequenced, including *CFH* and *CFI*, in a clinical diagnostic setting at the Laboratory of Genome Diagnostics (Radboud University Medical Center) using Sanger sequencing or next-generation sequencing (Illumina NextSeq 500) after enrichment with single-molecule molecular inversion probes. All genetic data were filtered for rare protein-altering or splice-site variants in *CFH* and *CFI*. In total, 379 individuals carried rare *CFH* or *CFI* variants, of whom 241 were affected by AMD (139 *CFH* carriers, 101 *CFI* carriers, and 1 *CFH* and *CFI* carrier). All rare variants in *CFH* and *CFI* were identified by at least 2 different genotyping platforms. If a rare variant in *CFH* or *CFI* was identified by 1 genotyping platform, it was confirmed using Sanger sequencing. For all noncarriers included in this study, whole-exome sequencing and/or single-molecule molecular inversion probe data were available to ensure that they did not carry any rare variants in *CFH, CFI, C3, C9*, or complement factor B. Genetic risk scores (GRSs) were assessed for all carriers and noncarriers based on both the 45 common AMD-associated variants and all the 52 AMD-associated variants^5^ and were calculated as previously described.^20^

### Statistical Analysis

General characteristics of the carriers and noncarriers were presented as means with standard deviations and proportions with percentages. Differences in general characteristics were analyzed using independent samples *t* tests for parametric distributions and χ^2^ tests for proportions. Genetic risk scores were compared using independent samples *t* tests. Phenotypic characteristics between carriers and noncarriers were compared using univariable generalized estimating equations binary logistic regression analyses to account for the fellow eye. The lowest category of each variable was used as the reference category. Results are reported as odds ratios (ORs), with corresponding confidence intervals (CI). In addition, we analyzed the *CFH* and *CFI* carriers separately to determine whether some characteristics are more strongly associated with either *CFH* or *CFI* carriership. Finally, we categorized the rare *CFH* and *CFI* variants into benign, likely benign, variant of uncertain significance, likely pathogenic, or pathogenic, based on functional studies to determine whether pathogenic variants are associated with a more severe phenotype (Table S1, available at www.ophthalmologyscience.org).^7^^,^^21^, ^22^, ^23^, ^24^, ^25^, ^26^, ^27^, ^28^, ^29^, ^30^, ^31^, ^32^, ^33^, ^34^, ^35^, ^36^, ^37^, ^38^, ^39^, ^40^, ^41^, ^42^, ^43^, ^44^, ^45^, ^46^, ^47^, ^48^, ^49^ In the subanalyses of rare pathogenic variants only, the reference category of the 2 variables predominant drusen type (reference category: hard drusen) and largest drusen size (reference category: drusen < C0) was not present in any eye of any carrier, resulting in failure of the model for those 2 variables in the specific subanalyses. Because Firth correction is not available in generalized estimating equations models, we used a fictive case in these subanalyses for the 2 aforementioned variables. We applied a Bonferroni correction for multiple testing in all analyses, and *P* values < 0.005 (α = 0.05/10) were considered statistically significant. All analyses were performed using SPSS, version 22 (IBM Corp).

## Results

### Cohort Description

**Figure 1:**
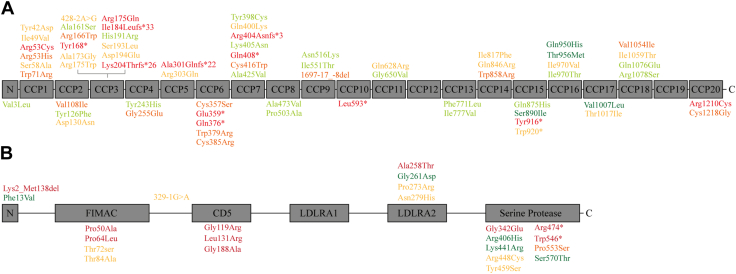
Genetic CFH and CFI variants included in this study. Rare genetic variants identified in the complement factor H and complement factor I genes in patients with age-related macular degeneration. Variants are organized according to their locations in the protein domains of factor H (**A**) and factor I (**B**). Each variant is categorized in one of the following pathogenicity classes based on literature (references are provided in Table S1, available at https://www.ophthalmologyscience.org); benign (indicated in green), likely benign (indicated in light green), variant of uncertain significance (indicated in yellow), likely pathogenic (indicated in orange), or pathogenic (indicated in red). C = C-terminus; CCP = complement control protein (indicated by numbers); CD5 = cluster of differentiation 5; FIMAC = factor I membrane attack complex; LDLRA1/LDLRA2 = low-density lipoprotein receptor A (1 and 2); N = N-terminus.

In total, 462 eyes of 234 carriers of rare variants in *CFH* and *CFI* and 468 eyes of 234 noncarriers were eligible for analyses. General characteristics of both study groups are outlined in Table 2. In total, 134 carriers of rare variants in *CFH* were identified, including 67 unique *CFH* variants, and 100 carriers of rare variants in *CFI* were identified, including 25 unique *CFI* variants (Fig 1; Table S1, available at www.ophthalmologyscience.org).

**Table 2:** General Characteristics of the Study Cohorts

Characteristic	Noncarriers (n = 234)	*CFH* and *CFI* Rare Variant Carriers (n = 234)	*P* Value
Age, y, mean (SD)	70.8 (12.4)	70.7 (12.5)	0.96
Gender, no. (%)			0.93
Male	93 (39.7)	92 (39.3)	
Female	141 (60.3)	142 (60.7)	
Smoking status, no. (%)			0.71
Never smoked	80 (39.2)	81 (40.5)	
Former smoker	100 (49.0)	91 (45.5)	
Current smoker	24 (11.8)	28 (14.0)	
Family history of AMD, no. (%)∗			0.02
Yes	64 (36.4)	86 (53.4)	
No	112 (63.6)	75 (46.6)	
Genetic risk score, mean (SD)	1.38 (1.24)	1.45 (1.35)	0.59
Visual acuity best eye, decimal, mean (SD)†	0.88 (0.41)	0.79 (0.36)	0.07

General characteristics of the study cohorts. Differences between *CFH* and *CFI* rare variant carriers and noncarriers were compared by using independent samples *t* tests for continuous variables and χ^2^ tests for nominal variables. AMD = age-related macular degeneration; *CFH* = complement factor H; *CFI* = complement factor I; SD = standard deviation.

∗ In the comparison of a positive family history of AMD between carriers and noncarriers (based on self-reported questionnaires), only the unrelated individuals were included (37 of 374 [9.9%] among the individuals with available information on family history of AMD were related, and therefore not included in this comparison).

† Only for n = 250 (100 noncarriers and 150 rare variant carriers), visual acuity data were available.

### Phenotype of Patients with AMD Carrying Rare Variants in CFH and CFI

First, we compared AMD disease stage between carriers and noncarriers (Table 3, part I, and Fig S2A, available at www.ophthalmologyscience.org) and observed intermediate AMD and GA more often in eyes of carriers of rare variants in *CFH* and *CFI* compared with the noncarriers (OR, 2.9; 95% CI, 1.8–4.6; OR, 2.4; 95% CI, 1.5–3.9, respectively) (Table 3, part I, section A). This observation did not change after stratifying the group into *CFH* carriers and *CFI* carriers only. In addition, CNV and mixed GA and CNV were also more prevalent in carriers of rare variants in *CFH*, although less pronounced (Table 3, part I, sections B1 and B2).

**Table 3:** Differences in Phenotypic Characteristics between Carriers of Variants in *CFH* and *CFI* and Noncarriers

Characteristics	Noncarriers (No. of Eyes = 468)	Section A			Section B1			Section B2		
		*CFH* and *CFI* Carriers (No. of Eyes = 462)	Odds Ratio∗ (95% CI)	*P* Value	*CFH* Carriers Only (No. of Eyes = 265)	Odds Ratio∗ (95% CI)	*P* Value	*CFI* Carriers Only (No. of Eyes = 197)	Odds Ratio∗ (95% CI)	*P* Value
Part I: AMD disease stage										
Disease stage				<0.001			<0.001			0.006
≤2 RC stage 2 (≤early AMD)	228 (48.7)	150 (32.5)	1 (Reference)		74 (27.9)	1 (Reference)		76 (38.6)	1 (Reference)	
RC stage 3 (intermediate AMD)	52 (11.1)	98 (21.2)	2.9 (1.8–4.6)		57 (21.5)	3.4 (1.9–5.9)		41 (20.8)	2.4 (1.3–4.2)	
RC stage 4 (CNV)	96 (20.5)	91 (19.7)	1.4 (0.9–2.2)		56 (21.1)	1.8 (1.1–2.9)		35 (17.8)	1.1 (0.6–1.9)	
RC stage 4 (GA)	57 (12.2)	90 (19.5)	2.4 (1.5–3.9)		52 (19.6)	2.8 (1.6–5.0)		38 (19.3)	2.0 (1.1–3.6)	
RC stage 4 (mixed CNV and GA)	35 (7.5)	33 (7.1)	1.4 (0.8–2.6)		26 (9.8)	2.3 (1.2–4.5)		7 (3.6)	0.6 (0.2–1.6)	
Part II: Specific phenotypic characteristics										
Predominant drusen type in grid, no. (%)				<0.001†			<0.001†			0.08
Hard	23 (5.9)	4 (1.0)	1 (Reference)		2 (0.9)	1 (Reference)		2 (1.1)	1 (Reference)	
Soft < C1 (125 μm)	110 (28.4)	72 (17.6)	3.8 (1.3–11.1)		34 (14.7)	3.6 (0.8–16.1)		38 (21.3)	4.0 (0.9–16.7)	
Soft distinct	34 (8.8)	26 (6.3)	4.4 (1.4–13.8)		11 (4.7)	3.7 (0.9–15.2)		15 (8.4)	5.1 (1.0–25.4)	
Soft indistinct	202 (52.1)	281 (68.5)	8.0 (2.7–23.6)		173 (74.6)	9.8 (2.3–42.7)		108 (60.7)	6.1 (1.4–26.8)	
Reticular	19 (4.9)	27 (6.6)	8.2 (2.3–29.1)		12 (5.2)	7.3 (1.4–38.9)		15 (8.4)	9.1 (1.7–48.3)	
Crystalline drusen, no. (%)				0.58			0.45			0.93
Absent	459 (98.1)	450 (97.4)	1 (Reference)		257 (97.0)	1 (Reference)		193 (98.0)	1 (Reference)	
Present	9 (1.9)	12 (2.6)	1.4 (0.5–4.0)		8 (3.0)	1.6 (0.5–5.3)		4 (2.0)	1.1 (0.3–3.9)	
Extramacular drusen, no. (%)				0.19			0.07			0.84
Absent	114 (24.9)	92 (20.4)	1 (Reference)		46 (17.8)	1 (Reference)		46 (24.0)	1 (Reference)	
Present	343 (75.1)	359 (79.6)	1.3 (0.9–1.9)		213 (82.2)	1.5 (1.0–2.5)		146 (76.0)	1.1 (0.6–1.7)	
Drusen nasal of the optic disc, no. (%)				0.90			0.96			0.86
Absent	425 (90.8)	421 (91.1)	1 (Reference)		241 (90.9)	1 (Reference)		180 (91.4)	1 (Reference)	
Present	43 (9.2)	41 (8.9)	1.0 (0.5–1.7)		24 (9.1)	1.0 (0.5–1.9)		17 (8.6)	0.9 (0.4–2.0)	
Largest drusen size in grid, no. (%)				<0.001†			<0.001†			0.03
<C0 (63 μm)	23 (5.9)	4 (1.0)	1 (Reference)		2 (0.9)	1 (Reference)		2 (1.1)	1 (Reference)	
<C1 (125 μm)	132 (34.1)	99 (24.3)	4.3 (1.5–12.5)		48 (20.9)	4.2 (1.0–17.6)		51 (28.8)	4.4 (1.1–18.7)	
<C2 (250 μm)	150 (38.8)	147 (36.1)	5.6 (1.9–16.8)		83 (36.1)	6.4 (1.4–28.0)		64 (36.2)	4.9 (1.1–21.7)	
≥C2 (250 μm)	65 (16.8)	129 (31.7)	11.4 (3.7–35.0)		84 (36.5)	14.9 (3.3–66.5)		45 (25.4)	8.0 (1.8–36.2)	
Reticular drusen	17 (4.4)	28 (6.9)	9.5 (2.6–34.1)		13 (5.7)	8.8 (1.6–47.5)		15 (8.5)	10.1 (1.9–54.5)	
Drusen area (%) in grid, no. (%)				<0.001†			<0.001†			0.001†
0%–10%	356 (76.2)	255 (55.8)	1 (Reference)		141 (53.6)	1 (Reference)		114 (58.8)	1 (Reference)	
10%–25%	62 (13.3)	104 (22.8)	2.3 (1.6–3.5)		70 (26.6)	2.9 (1.8–4.5)		34 (17.5)	1.7 (1.02–2.9)	
25%–50%	33 (7.1)	77 (16.8)	3.3 (1.9–5.5)		42 (16.0)	3.2 (1.8–5.8)		35 (18.0)	3.3 (1.8–6.2)	
>50%	16 (3.4)	21 (4.6)	1.8 (0.8–4.1)		10 (3.8)	1.6 (0.6–4.0)		11 (5.7)	2.1 (0.8–5.5)	
Increased pigment, no. (%)				0.05			0.12			0.04
No pigment	244 (54.0)	209 (46.2)	1 (Reference)		114 (44.2)	1 (Reference)		95 (49.0)	1 (Reference)	
<C1 (125 μm)	2 (0.4)	10 (2.2)	5.8 (1.3–26.9)		4 (1.6)	4.3 (0.8–23.8)		6 (3.1)	7.7 (1.5–38.5)	
<C2 (250 μm)	25 (5.5)	34 (7.5)	1.6 (0.9–2.8)		16 (6.2)	1.4 (0.7–2.8)		18 (9.3)	1.8 (1.0–3.6)	
≥C2 (250 μm)	181 (40.0)	199 (44.0)	1.3 (0.9–1.8)		124 (48.1)	1.5 (1.0–2.2)		75 (38.7)	1.1 (0.7–1.6)	
RPE degeneration, no. (%)				0.12			0.10			0.16
No RPE degeneration	349 (77.2)	308 (69.8)	1 (Reference)		172 (69.1)	1 (Reference)		136 (70.8)	1 (Reference)	
<C2 (250 μm)	18 (4.0)	24 (5.4)	1.5 (0.8–2.9)		9 (3.6)	1.0 (0.4–2.3)		15 (7.8)	2.1 (1.01–4.5)	
<5× C2 (250 μm)	50 (11.1)	75 (17.0)	1.7 (1.1–2.6)		48 (19.3)	1.9 (1.2–3.1)		27 (14.1)	1.4 (0.8–2.5)	
<Central grid	16 (3.5)	19 (4.3)	1.4 (0.7–2.8)		10 (4.0)	1.3 (0.6–2.9)		9 (4.7)	1.4 (0.6–3.6)	
≥Central grid	19 (4.2)	15 (3.4)	0.9 (0.4–2.0)		10 (4.0)	1.1 (0.4–2.7)		5 (2.6)	0.7 (0.2–1.9)	

Phenotypic characteristics of patients with AMD carrying rare *CFH* and *CFI* variants and AMD noncarriers. In the main analysis, the carrier group included *CFH* and *CFI* rare variant carriers combined (N = 462; section A). In the subanalysis, phenotypic characteristics were compared between *CFH* rare variant carriers only (N = 265) and noncarriers, and between *CFI* rare variant carriers only (N = 197) and noncarriers (section B). The total number of eyes in each of the groups is indicated in the headings of the table. Sometimes the numbers in the table do not add up to the total number of eyes indicated in the headings. In these cases, specific characteristics were not applicable or could not be graded in some eyes. AMD = age-related macular degeneration; *CFH* = complement factor H; *CFI* = complement factor I; CI = confidence interval; CNV = choroidal neovascularization; GA = geographic atrophy; RC = Rotterdam Classification; RPE = retinal pigment epithelium.

∗ Odds ratios result from univariable generalized estimating equations binary logistic regression analyses.

† Phenotypic characteristics that remain significant after Bonferroni correction for multiple testing.

As a second step, we analyzed individual AMD features on CFP in both groups (Table 3, part II, section A and Fig S2B–D). Predominant drusen type, largest drusen size, and drusen area in the ETDRS grid were significantly associated with carriership of a rare variant in *CFH* or *CFI* (*P* < 0.001). With regard to the predominant drusen type in the ETDRS grid, we observed that especially soft indistinct drusen (OR [95% CI], 8.0 [2.7–23.6]) and reticular pseudodrusen (RPD) (OR [95% CI], 8.2 [2.3–29.1]) were more often seen in carriers compared with noncarriers. Increasing ORs (95% CIs) were observed with increasing drusen size in the ETDRS grid in carriers compared with noncarriers, ranging from 4.3 (1.5–12.5) to 5.6 (1.9–16.8) to 11.4 (3.7–35.0) for largest drusen size of <125, <250, and ≥250 μm, respectively. Furthermore, carriers of rare variants in *CFH* and *CFI* presented with a larger macular area covered with drusen compared with noncarriers (*P* < 0.001).

We repeated the analyses for carriers of rare variants in *CFH* and *CFI* separately, to determine whether some characteristics were more strongly associated with either *CFH* or *CFI* carriership (Table 3, part II, sections B1 and B2). Predominant drusen type (*P* < 0.001), largest drusen size (*P* < 0.001), and drusen area (*P* < 0.001) were associated with carriership of a rare variant in *CFH*. Drusen area (*P* = 0.001) was the only feature associated with carriership of a rare variant in *CFI*, and a trend was observed for largest drusen size (*P* = 0.03) and RPE pigmentation (*P* = 0.04).

### Subanalyses Based on Pathogenicity of the Variants

Next, we repeated the analyses by including pathogenic rare variants only (Table 4, part I). For the disease stages, we observed the same directions of effect. However, the associations of intermediate AMD and GA with carriership of rare variants in *CFH* had much larger effect sizes (OR, 5.1; 95% CI, 1.9–13.7; OR, 7.4; 95% CI, 3.0–18.0, respectively). Regarding the specific phenotypic characteristics (Table 4, part II), we observed significant associations of predominant drusen type, largest drusen size, and drusen area with carriership of a rare pathogenic variant in *CFH* or *CFI* (*P* < 0.001 for all), and effect sizes of all associations were larger compared with the analyses with all rare variants. Carriers of pathogenic variants were more likely to have RPE pigmentation (*P* = 0.02) and RPE degeneration (*P* = 0.03) compared with noncarriers.

**Table 4:** Differences in Phenotypic Characteristics between Carriers of Pathogenic Variants in *CFH* and *CFI* and Noncarriers

Characteristics	Noncarriers (No. of Eyes = 468)	Section A			Section B1			Section B2		
		CFH and CFI Carriers (No. of Eyes = 151)	Odds Ratio∗ (95% CI)	*P* Value	CFH Carriers Only (No. of Eyes = 66)	Odds Ratio∗ (95% CI)	*P* Value	CFI Carriers Only (No. of eyes = 85)	Odds Ratio∗ (95% CI)	*P* Value
Part I: AMD disease stage										
Disease stage				<0.001			<0.001			0.01
≤2 RC stage 2 (≤early AMD)	228 (48.7)	46 (30.5)	1 (Reference)		13 (19.7)	1 (Reference)		33 (38.8)	1 (Reference)	
RC stage 3 (intermediate AMD)	52 (11.1)	34 (22.5)	3.2 (1.7–6.2)		15 (22.7)	5.1 (1.9–13.7)		19 (22.4)	2.5 (1.2–5.4)	
RC stage 4 (CNV)	96 (20.5)	21 (13.9)	1.1 (0.6–2.1)		8 (12.1)	1.5 (0.5–4.1)		13 (15.3)	0.9 (0.4–2.1)	
RC stage 4 (GA)	57 (12.2)	43 (28.5)	3.7 (2.0–7.0)		24 (36.4)	7.4 (3.0–18.0)		19 (22.4)	2.3 (1.1–5.0)	
RC stage 4 (mixed CNV and GA)	35 (7.5)	7 (4.6)	1.0 (0.3–2.9)		6 (9.1)	3.0 (0.8–11.3)		1 (1.2)	0.2 (0.03–1.5)	
Part II: Specific phenotypic characteristics										
Predominant drusen type in grid, no. (%)‡				<0.001†			0.002†			0.05
Hard	23 (5.9)	0 (0.0)	1 (Reference)		0 (0.0)	1 (Reference)		0 (0.0)	1 (Reference)	
Soft < C1 (125 μm)	110 (28.4)	15 (10.6)	3.1 (0.4–25.5)		5 (7.8)	1.0 (0.1–10.0)		10 (12.8)	2.1 (0.2–17.7)	
Soft distinct	34 (8.8)	7 (4.9)	4.7 (0.5–44.1)		0 (0.0)	0.7 (0.0–11.4)		7 (9.0)	4.7 (0.5–44.1)	
Soft indistinct	202 (52.1)	113 (79.6)	12.9 (1.7–97.3)		57 (89.1)	6.5 (0.8–49.9)		56 (71.8)	6.4 (0.8–49.0)	
Reticular	19 (4.9)	7 (4.9)	8.5 (0.9–84.2)		2 (3.1)	2.4 (0.1–41.7)		5 (6.4)	6.1 (0.6–65.0)	
Crystalline drusen, no. (%)				0.66			0.31			0.65
Absent	459 (98.1)	147 (97.4)	1 (Reference)		63 (95.4)	1 (Reference)		84 (98.8)	1 (Reference)	
Present	9 (1.9)	4 (2.6)	1.4 (0.3–6.1)		3 (4.5)	2.4 (0.4–13.5)		1 (1.2)	0.6 (0.1–5.1)	
Extramacular drusen, no. (%)				0.24			0.04			0.96
Absent	114 (24.9)	28 (18.8)	1 (Reference)		7 (10.6)	1 (Reference)		21 (25.3)	1 (Reference)	
Present	343 (75.1)	121 (81.2)	1.4 (0.8–2.6)		59 (89.4)	2.8 (1.03–7.6)		62 (74.7)	1.0 (0.5–2.0)	
Drusen nasal of the optic disc, no. (%)				0.58			0.15			0.65
Absent	425 (90.8)	134 (88.7)	1 (Reference)		55 (83.3)	1 (Reference)		79 (92.9)	1 (Reference)	
Present	43 (9.2)	17 (11.3)	1.3 (0.6–2.8)		11 (16.7)	2.0 (0.8–5.0)		6 (7.1)	0.8 (0.2–2.6)	
Largest drusen size in grid, no. (%)‡				<0.001†			<0.001†			0.006†
<C0 (63 μm)	23 (5.9)	0 (0.0)	1 (Reference)		0 (0.0)	1 (Reference)		0 (0.0)	1 (Reference)	
<C1 (125 μm)	132 (34.1)	19 (13.6)	3.3 (0.4–26.3)		5 (7.9)	0.9 (0.1–8.3)		14 (18.2)	2.4 (0.3–19.8)	
<C2 (250 μm)	150 (38.8)	53 (37.9)	8.1 (1.1–62.3)		23 (36.5)	3.5 (0.4–28.2)		30 (39.0)	4.6 (0.6–35.9)	
≥C2 (250 μm)	65 (16.8)	59 (42.1)	20.9 (2.7–161.8)		31 (49.2)	11.0 (1.4–87.3)		28 (36.4)	9.9 (1.2–78.7)	
Reticular drusen	17 (4.4)	9 (6.4)	12.2 (1.3–116.3)		4 (6.3)	5.4 (0.4–65.4)		5 (6.5)	6.8 (0.6–73.2)	
Drusen area (%) in grid, no. (%)				<0.001†			<0.001†			<0.001†
0%–10%	357 (76.3)	63 (42.6)	1 (Reference)		24 (36.4)	1 (Reference)		39 (47.6)	1 (Reference)	
10%–25%	62 (13.2)	35 (23.6)	3.2 (1.8–5.6)		21 (31.8)	5.0 (2.3–10.9)		14 (17.1)	2.1 (1.01–4.2)	
25%–50%	33 (7.1)	37 (25.0)	6.3 (3.3–12.2)		17 (25.8)	7.6 (3.1–18.7)		20 (24.4)	5.5 (2.5–12.1)	
>50%	16 (3.4)	13 (8.8)	4.6 (1.8–11.5)		4 (6.1)	3.7 (0.9–15.4)		9 (11.0)	5.1 (1.9–14.1)	
Increased pigment, no. (%)				0.02			0.32			0.006†
No pigment	244 (54.0)	70 (47.0)	1 (Reference)		26 (40.0)	1 (Reference)		44 (52.4)	1 (Reference)	
<C1 (125 μm)	2 (0.4)	7 (4.7)	12.2 (2.5–59.6)		1 (1.5)	4.7 (0.4–54.7)		6 (7.1)	16.6 (3.3–83.6)	
<C2 (250 μm)	25 (5.5)	10 (6.7)	1.4 (0.6–3.2)		6 (9.2)	2.3 (0.7–6.9)		4 (4.8)	0.9 (0.3–2.6)	
≥C2 (250 μm)	181 (40.0)	62 (41.6)	1.2 (0.7–2.0)		32 (49.2)	1.7 (0.8–3.4)		30 (35.7)	0.9 (0.5–1.8)	
RPE degeneration, no. (%)				0.03			0.01			0.47
No RPE degeneration	349 (77.2)	101 (69.2)	1 (Reference)		40 (62.5)	1 (Reference)		61 (74.4)	1 (Reference)	
<C2 (250 μm)	18 (4.0)	9 (6.2)	1.7 (0.8–3.9)		3 (4.7)	1.5 (0.4–4.9)		6 (7.3)	1.9 (0.7–4.9)	
<5× C2 (250 μm)	50 (11.1)	26 (17.8)	1.8 (1.0–3.3)		14 (21.9)	2.4 (1.2–5.0)		12 (14.6)	1.4 (0.6–3.2)	
<Central grid	16 (3.5)	9 (6.2)	1.9 (0.8–4.8)		6 (9.4)	3.3 (1.2–8.8)		3 (3.7)	1.1 (0.2–5.2)	
≥Central grid	19 (4.2)	1 (0.7)	0.2 (0.0–1.4)		1 (1.6)	0.5 (0.1–3.6)		0 (0.0)	0.3 (0.0–2.4)	

Phenotypic characteristics of patients with AMD carrying rare pathogenic *CFH* and *CFI* variants and AMD noncarriers. In the main analysis, the carrier group included *CFH* and *CFI* rare variant carriers combined (N = 151; section A). In the subanalysis, phenotypic characteristics were compared between *CFH* rare variant carriers only (N = 66) and noncarriers, and between *CFI* rare variant carriers only (N = 85) and noncarriers (section B). The total number of eyes in each of the groups is indicated in the headings of the table. Sometimes the numbers in the table do not add up to the total number of eyes indicated in the headings. In these cases, specific characteristics were not applicable or could not be graded in some eyes.

AMD = age-related macular degeneration; CFH = complement factor H; CFI = complement factor I; CI = confidence interval; CNV = choroidal neovascularization; GA = geographic atrophy; RC = Rotterdam Classification; RPE = retinal pigment epithelium.

∗ Odds ratios result from univariable generalized estimating equations binary logistic regression analyses.

† Phenotypic characteristics that remain significant after Bonferroni correction for multiple testing.

‡ In all 3 subanalyses (*CFH* and *CFI* rare variant carriers vs. noncarriers, *CFH* rare variant carriers only vs. noncarriers, *CFI* rare variant carriers vs. noncarriers), the reference category of the 2 variables drusen type (reference category: hard drusen) and drusen size (reference category: drusen < C0) was not present in any eye of any carrier, resulting in failure of the model for those 2 variables. As in generalized estimating equations models, Firth correction is not available; therefore, we used a fictive case only in the subanalyses for the 2 aforementioned variables.

**Figure 3:**
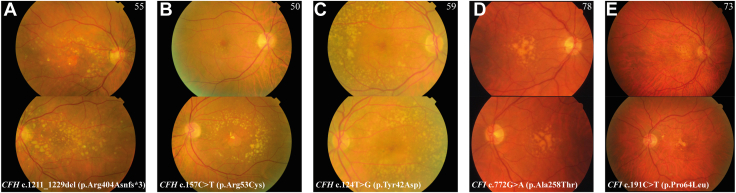
Color fundus photographs (CFPs) of patients with age-related macular degeneration carrying rare complement factor H and complement factor I variants. Examples of CFP of patients with age-related macular degeneration carrying rare genetic variants in the complement factor H gene (**A–C**) or in the complement factor I genes (**D, E**). Age at time of examination is indicated in the right corner of each panel. At the bottom of each panel, the specific rare variant is given. Upper CFP in each panel represents the patients’ right eyes, and the bottom CFP in each panel represents the patients’ left eyes. Patient A and B both carry a pathogenic rare variant in the complement factor H gene and are both affected by geographic atrophy in 1 eye at a young age. Large (≥250 μm), soft, indistinct drusen are detected in the macula of both eyes of patient A and the left eye of patient B. The fundus of the patient C was characterized by an extensive amount of large, soft, indistinct drusen located predominantly outside the macula. Color fundus photograph of both eyes showed high symmetry, which is also the case for patient D. In patient D, a large area of the macula was covered by large, soft, indistinct, confluent drusen. The rare variant in complement factor I in this patient was reported to lead to skipping of exon 5, resulting in reduced factor I or factor I deficiency.^39^^,^^46^ Patient E was affected by advanced age-related macular degeneration, and reticular pseudodrusen in both eyes were observed.

As a last step, we analyzed the carriers of rare pathogenic variants in *CFH* and *CFI* separately (Table 4, part II, sections B1 and B2). Predominant drusen type (*P* = 0.002), largest drusen size (*P* < 0.001), and drusen area (*P* < 0.001) were associated with carriership of rare pathogenic *CFH* variants, and a trend was observed for extramacular drusen (*P* = 0.04) and RPE degeneration (*P* = 0.01). Largest drusen size (*P* = 0.006), drusen area (*P* < 0.001), and RPE pigmentation (*P* = 0.006) were associated with carriership of rare pathogenic *CFI* variants. In Figure 3,^39^^,^^46^ examples of 5 patients with AMD are shown, including fundus features that were characteristic for carriership of a rare (pathogenic) variant in *CFH* or *CFI*.

Because the prevalence of GA and CNV increases with age, and the fact that a severe phenotype could also be caused by high risk alleles, we compared age and GRSs between carriers and noncarriers within the different subgroups (Tables S5 and S6, available at www.ophthalmologyscience.org). Carriers of rare pathogenic variants in *CFH* were younger (64.3 vs. 70.8 years, *P* < 0.001), whereas carriers of rare pathogenic variants in *CFI* were a bit older (72.4 vs. 70.8 years, *P* = 0.28) compared with noncarriers. Genetic risk scores did not differ between carriers of rare (pathogenic) variants in *CFH* and noncarriers (1.22 vs. 1.38, *P* = 0.25 for all *CFH* carriers vs. noncarriers; 1.01 vs. 1.38, *P* = 0.18 for all pathogenic *CFH* carriers vs. noncarriers), whereas carriers of rare (pathogenic) variants in *CFI* had (a trend toward) a higher GRS compared with noncarriers (1.74 vs. 1.38; *P* = 0.02 for all *CFI* carriers vs. noncarriers; 2.10 vs. 1.38, *P* = 0.002 for pathogenic *CFI* carriers vs. noncarriers). Because 7/52 variants in the GRS are rare variants with relatively large effect sizes, including the *CFI* c.355G>A (p.Gly119rg) variant that was present in 20 of our patients in the *CFI* cohort, we also compared the GRS between the groups by including only the 45 common variants. We observed a trend toward a lower GRS in carriers of rare pathogenic variants in *CFH* compared with noncarriers (0.83 vs. 1.41, *P* = 0.03). Genetic risk scores of the other carrier groups did not differ compared with noncarriers.

## Discussion

In this study, we compared phenotypic characteristics on CFP between patients with AMD with and without rare variants in *CFH* and *CFI* and found that patients with AMD carrying rare variants in *CFH* and *CFI* are characterized by a more severe drusen phenotype, including large, soft indistinct drusen; RPD, a large area of the macula covered by drusen; and a higher frequency of intermediate AMD and GA. Subgroup analysis showed that these characteristics were mainly driven by the carriership of rare variants in *CFH*, except for drusen area, intermediate AMD, and GA, which were also associated with *CFI* rare variants. Analysis of rare pathogenic variants only revealed that almost all associations had larger effect sizes, especially the association of GA with carriership of rare pathogenic variants in *CFH*.

Despite the limited number of studies that focus on this subgroup of rare variant carriers, some of the identified phenotypic features in our study overlapped with that of other studies. A larger macular drusen area has previously been associated with carriership of a rare variant in *CFH*.^15^^,^^50^ Reticular pseudodrusen were also reported in 15 of 21 patients with AMD with GA who carried a rare variant in *CFI* in combination with low serum factor I levels.^51^ Furthermore, Saksens et al^52^ found an association between RPD and specific rare variants (*CFI* p.Gly119Arg, *C3* p.Lys155Gln, and *C9* p.Pro167Ser). In our study, the prevalence of RPD might be underestimated because grading was based on CFPs, whereas other image modalities, such as spectral domain-OCT and infrared imaging, are more sensitive for the detection of RPD.^53^

We were not able to replicate the association of drusen located nasal of the optic disc and crystalline drusen in carriers of rare (pathogenic) variants in *CFH*.^15^ Although in the current study the frequencies or drusen nasal of the optic disc and crystalline drusen were a bit higher in carriers of rare pathogenic variants in *CFH* compared with noncarriers, this difference was not significant. However, in the current study, these features were not part of the standard grading protocol, and therefore, only noted in the comment section according to the discretion of the graders, meaning that these features may be underestimated.

In a large population-based study, multiple fundus features, including a drusen area larger than 375 μm and drusen type (soft, indistinct, and RPD), were reported to be strongly associated with risk for progression to GA.^54^ This might explain the higher prevalence of GA in our rare variant carriers, in whom we observe a more severe drusen phenotype. This association is in line with a recent study by Seddon et al,^55^ showing a higher risk of progression to GA in patients with AMD carrying rare pathogenic variants in *CFI*. In addition, the p.Arg1210Cys variant in *CFH* was also associated with advanced AMD, and in particular GA.^50^ The higher prevalence of GA in carriers of rare variants in *CFH* and *CFI* compared with noncarriers suggests that these patients may be suitable candidates for treatment with complement inhibitors.

A possible explanation for the more severe drusen phenotype in our rare variant carriers might be related to the stronger dysregulation of the complement system in these patients. Complement components, such as C3 and C5, are components of conventional drusen.^56^ Additionally, RPD contain immune cells and photoreceptor outer segments, and are rich in vitronectin. This latter component interacts with complement proteins and inhibits the terminal cytolytic part of the complement pathway.^57^ One might argue that in patients carrying rare genetic variants in complement-related genes, such as *CFH* and *CFI*, accumulation of complement components may be more severe or may even occur at an earlier age.

The arrival of complement-inhibiting therapies for AMD raises the question of which patients would respond best to these treatments. It might be reasonable to suggest that these are patients with a rare variant in one of the complement genes (especially those resulting in reduced protein levels or reduced protein function), patients with a high complement genetic risk score, or patients with high systemic complement activation levels. Proper stratification of patients into subgroups is therefore important for the selection of patients for clinical trials. Based on fundus features, in combination with an early age of onset and a positive family history of AMD,^47^^,^^52^^,^^58^^,^^59^ ophthalmologists could identify patients with a high likelihood of carrying rare variants in *CFH* or *CFI* more easily. Additional genetic testing in this subgroup with high clinical suspicion would confirm carriership of rare *CFH* and *CFI* variants. Of note, *CFH* and *CFI* variants are associated with intermediate AMD and GA. Patients with GA have the largest unmet need because treatment options are limited, which has increased interest in the ophthalmology field to identify interventions that can prevent progression from intermediate AMD to advanced AMD.

The ultimate aspiration in AMD is to prevent or slow down the progression from the intermediate to the advanced stage rather than focusing merely on interventions in the presence of stages. This remains an active area of research. Given the association between rare variants in *CFH* and *CFI* with a more severe drusen phenotype and a higher prevalence of GA, we propose that this subgroup of patients represents a promising target for future clinical trials investigating complement inhibitors in earlier stages of AMD. Carriers of rare *CFH* variants may, in particular, be candidates for treatment since, because of their younger age, they are burdened by many more years of vision loss.

### Strengths and Limitations

One of the major strengths of this study is the large number of carriers of rare variants in *CFH* and *CFI*. Although 34% of *CFH* carriers overlapped, because this study is an extension of the study by Kersten et al,^15^ the substantial increase in sample size of *CFH* carriers and the addition of *CFI* carriers allowed for a detailed analysis of phenotypic features. Because the objective of our study was to describe the phenotypic characteristics of carriers of rare *CFH* and *CFI* variants, we did not exclude the 45 *CFH* carriers from the study of Kersten et al.^15^ Excluding these carriers would provide an incomplete picture of the *CFH* phenotype.

Although we used a detailed grading protocol for AMD features within the ETDRS grid, it was limited in the detailed grading of drusen outside the ETDRS grid (e.g., drusen nasal to the optic disc, extramacular drusen), which we believe are important phenotypic features to include when studying AMD. Second, the addition of other imaging techniques would allow for better detection of specific phenotypic characteristics (e.g., OCT, infrared imaging for RPD, peripheral fundus photographs, and, although more invasive, fluorescein angiography for cuticular drusen).

Furthermore, the growing number of functional studies on rare variants allows for stratification of rare variants that were previously classified as “variant of unknown significance” into (likely) pathogenic or (likely) benign.^23^^,^^28^, ^29^, ^30^^,^^33^^,^^48^^,^^49^ However, the pathogenicity of part of the rare variants in our study remains unknown so far. The more severe phenotype in carriers of pathogenic rare variants in our study underlines the importance of functional characterization as this improves risk assessment.

In this study, the carrier and noncarrier groups were matched on age because age is an important risk factor for progression, and thus will have an effect on the disease. Taking into account the important role of genetic factors, one might consider matching on GRS as well. However, we chose not to match on GRS because we hypothesized that our rare variant carriers would have a lower GRS compared with the noncarries, as the risk conferred by a high GRS is outweighed by the impact of the rare variant, therefore rendering the GRS of less importance in the rare variant carriers. Consequently, matching on GRS might have resulted in a noncarrier cohort with a lower GRS than expected for the general AMD population. This theory is supported by our data in Table S6 (available at www.ophthalmologyscience.org), where we show that the GRS based on the 45 common variants that are included was similar between our *CFI* carriers and the noncarriers, but lower in our *CFH* carriers compared with our noncarriers. As we have previously shown, a higher GRS is correlated with a more advanced disease stage (PMID: 32717343 and 33253757).^20^^,^^60^ Matching on GRS would therefore likely have resulted in lower disease stages among the noncarriers, leading to a noncarrier cohort that is not representative of the general AMD population.

Although the primary focus of this study was on phenotypic characteristics, we also explored visual acuity as a functional parameter. No statistically significant difference in visual acuity was observed between rare variant carriers and noncarriers. Future studies incorporating more sensitive functional measures, such as contrast sensitivity or dark adaptation testing, may provide additional insight into more subtle functional differences between these groups. Additionally, it would be worthwhile to study the phenotype of patients with AMD carrying rare variants in other complement genes, for example *C3*, especially due to the recent Food and Drug Administration approval of the C3 inhibitor pegcetacoplan.

In conclusion, patients with AMD carrying rare variants in *CFH* and *CFI* present with a more severe drusen phenotype and have a higher prevalence of GA, in particular those carrying rare pathogenic variants in *CFH*. Stratification of patients in subgroups is important for future studies to determine which patients benefit most from complement inhibition. Phenotypic characteristics of CFP, together with other characteristics associated with carriership of rare variants in *CFH* and *CFI*, such as an early age of onset and a positive family history of AMD, could aid ophthalmologists in identifying patients with AMD who are more likely to carry a rare variant in *CFH* or *CFI*.
