# Supplementary material for: Geographic Atrophy in Patients with Age-Related Macular Degeneration Is Associated with Rare Variants in Complement Factor H and Complement Factor I
Source: Ophthalmol Sci. 2026 Mar 27;6(6):101171. doi: 10.1016/j.xops.2026.101171 (PMC13218239; doi:10.1016/j.xops.2026.101171)
Supplement: Figure S2 [file mmc1.docx]

**Supplementary Figure 2.** Proportions of Phenotypic Characteristics in Carriers and Noncarriers


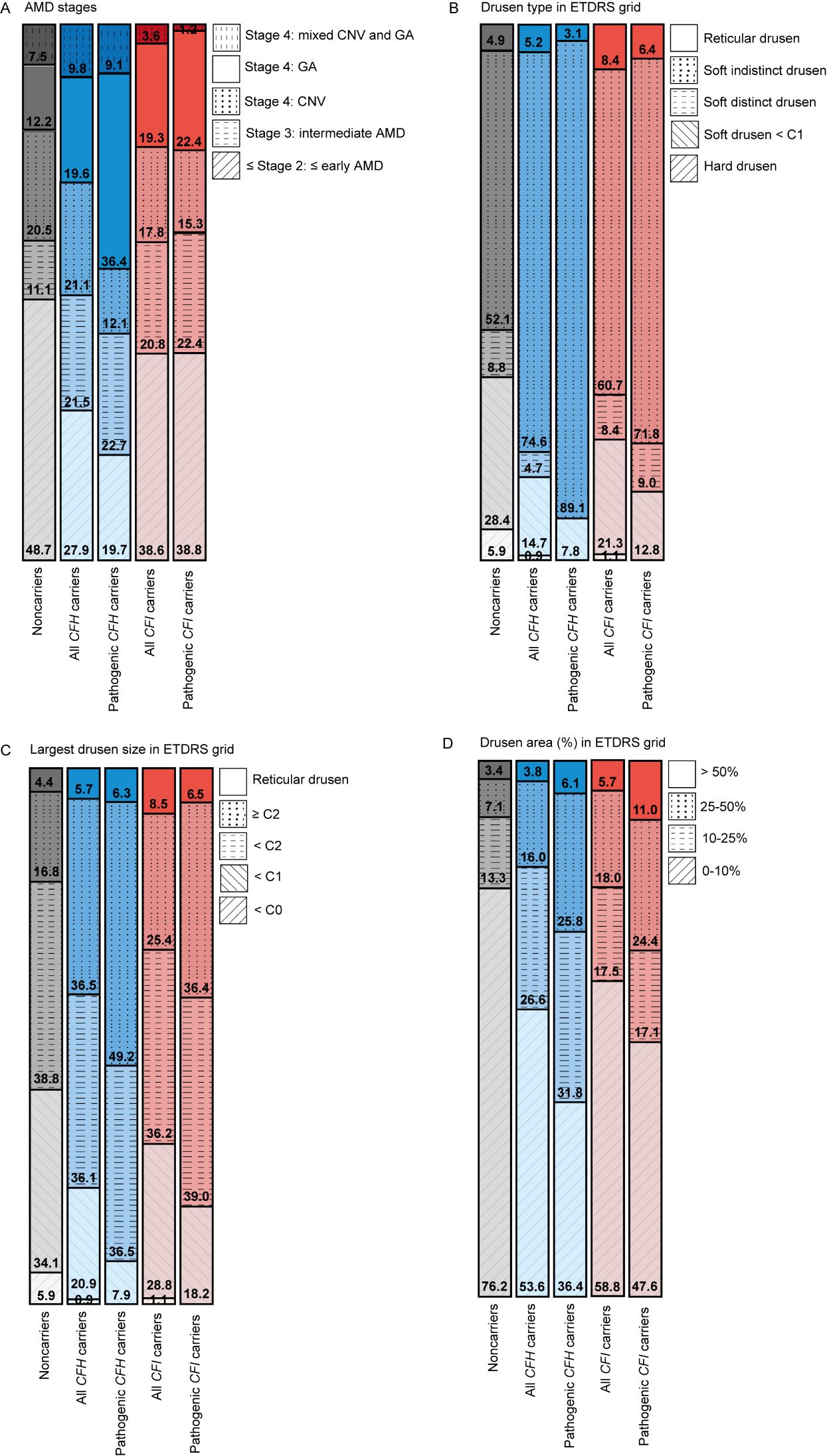


Proportions of AMD stages and several phenotypic characteristics in AMD patients carrying rare variants in CFH and/or CFI and AMD noncarriers. Numbers in the bars include the proportions. CFH = complement factor H; CFI = complement factor I; ETDRS = early treatment of diabetic retinopathy.
