# Supplementary material for: Geographic Atrophy in Patients with Age-Related Macular Degeneration Is Associated with Rare Variants in Complement Factor H and Complement Factor I
Source: Ophthalmol Sci. 2026 Mar 27;6(6):101171. doi: 10.1016/j.xops.2026.101171 (PMC13218239; doi:10.1016/j.xops.2026.101171)
Supplement: Table S1 [file mmc2.pdf]

Supplementary Table 1. Rare Genetic Variants in the *CFH* and *CFI* Genes Identified in AMD Patients

Gene	Genomic position	Nucleotide change	Protein change [a]	Minor allele frequency (%) [b]	No. of AMD patients	Pathogenicity category rare variant	References pathogenicity categories (functional studies)
**Carriers of a single rare *CFH* or *CFI* variant**							
CFH	196621254	7C>G	Leu3Val	0.03	2	Likely benign	De Jong et al, 2022
CFH	196642173	124T>G	Tyr42Asp	n/a	1	VUS	-
CFH	196642194	145A>G	Ile49Val	0.0009	1	VUS	De Jong et al, 2022
CFH	196642206	157C>T	Arg53Cys	0.004	1	Pathogenic	Yu et al, 2014; Merinero et al, 2018; Fakhouri et al, 2010; Servais et al, 2012
CFH	196642207	158G>A	Arg53His	n/a	1	Likely pathogenic	Pechtl et al, 2011
CFH	196642221	172T>G	Ser58Ala	0.03	1	VUS	Merinero et al, 2018; De Jong et al, 2022
CFH	196642260	211T>A	Trp71Arg	n/a	1	Likely pathogenic	De Jong et al, 2022
CFH	196643064	322G>A	Val108Ile	n/a	1	Likely pathogenic	De Jong et al, 2022
CFH	196645145	377A>T	Tyr126Phe	n/a	1	Likely benign	De Jong et al, 2022
CFH	196645156	388G>A	Asp130Asn	0.03	1	VUS	Servais et al, 2012
CFH	196646604	428-2A>G	-	n/a	1	VUS	-
CFH	196646674	496C>T	Arg166Trp	0.0009	1	Likely pathogenic	De Jong et al, 2022
CFH	196646682	504C>A	Tyr168*	n/a	1	Pathogenic	De Jong et al, 2022
CFH	196646696	518C>G	Ala173Gly	n/a	1	VUS	De Jong et al, 2022
CFH	196646701	523C>T	Arg175Trp	n/a	1	VUS	-
CFH	196646702	524G>A	Arg175Gln	n/a	9	Pathogenic	Geerlings et al, 2017; De Jong et al, 2022
CFH	196646727	550delA	Ile184Leufs*33	n/a	1	Pathogenic	De Jong et al, 2022
CFH	196646750	572A>G	His191Arg	n/a	1	Likely benign	De Jong et al, 2022
CFH	196646760	582C>A	Aps194Glu	0.0009	1	VUS	De Jong et al, 2022
CFH	196646782	607_610dupCCAA	Lys204Thrfs*26	n/a	3	Pathogenic	De Jong et al, 2022
CFH	196648860	727T>C	Tyr243His	n/a	1	Likely benign	De Jong et al, 2022
CFH	196648897	764G>A	Gly255Glu	0.004	2	Likely pathogenic	De Jong et al, 2022
CFH	196654303	901delG	Ala301Glnfs*22	n/a	4	Pathogenic [d]	-
CFH	196658654	1069T>A	Cys357Ser	n/a	1	Likely pathogenic	De Jong et al, 2022
CFH	196658660	1075G>T	Glu359*	n/a	1	Pathogenic	De Jong et al, 2022
CFH	196658711	1126C>T	Gln376*	n/a	1	Pathogenic	De Jong et al, 2022
CFH	196658720	1135T>C	Trp379Arg	n/a	1	Likely pathogenic	De Jong et al, 2022
CFH	196658738	1153T>C	Cys385Arg	n/a	1	Likely pathogenic	De Jong et al, 2022

Supplementary Table 1. Rare Genetic Variants in the *CFH* and *CFI* Genes Identified in AMD Patients (continued)

Gene	Genomic position	Nucleotide change	Protein change [a]	Minor allele frequency (%) [b]	No. of AMD patients	Pathogenicity category rare variant	References pathogenicity categories (functional studies)
CFH	196659226	1193A>G	Tyr398Cys	n/a	1	Likely benign	De Jong et al, 2022
CFH	196659231	1198C>A	Gln400Lys	0.02	3	VUS	De Jong et al, 2022; Dragon-Durey et al, 2004
CFH	196659243	1211_1229del	Arg404Asnfs*3	n/a	1	Pathogenic [d]	-
CFH	196659248	1215G>T	Lys405Asn	0.0009	1	Likely benign	De Jong et al, 2022
CFH	196659255	1222C>T	Gln408*	n/a	6	Pathogenic	De Jong et al, 2022
CFH	196659281	1248C>G	Cys416Trp	n/a	1	Likely pathogenic	De Jong et al, 2022
CFH	196659307	1274C>T	Ala425Val	0.002	1	Likely benign	De Jong et al, 2022
CFH	196682946	1418C>T	Ala473Val	0.01	2	Likely benign	De Jong et al, 2022
CFH	196683035	1507C>G	Pro503Ala	0.004	1	Likely benign	De Jong et al, 2022
CFH	196684751	1548T>A	Asn516Lys	0.04	3	Likely benign	De Jong et al, 2022
CFH	196684855	1652T>C	Ile551Thr	0.01	1	Likely benign	De Jong et al, 2022
CFH	196694231	1697-17_1697-8delATTTTACCTT	-	0.001	1	Likely pathogenic	De Jong et al, 2022
CFH	196694332	1778T>A	Leu593*	n/a	2	Pathogenic	De Jong et al, 2022
CFH	196695609	1883A>G	Gln628Arg	n/a	1	VUS	-
CFH	196695675	1949G>T	Gly650Val	0.04	4	Likely benign	De Jong et al, 2022
CFH	196697552	2313C>A	Phe771Leu	n/a	1	Likely benign	De Jong et al, 2022
CFH	196697568	2329A>G	Ile777Val	0.003	1	Likely benign	De Jong et al, 2022
CFH	196705989	2449A>T	Ile817Phe	n/a	1	VUS	-
CFH	196706077	2537A>G	Gln846Arg	n/a	1	VUS	-
CFH	196706112	2572T>A	Trp858Arg	n/a	5	Likely pathogenic	De Jong et al, 2022
CFH	196706633	2625G>C	Gln875His	n/a	1	Likely benign	De Jong et al, 2022
CFH	196706677	2669G>T	Ser890Ile	0.14	1	Benign	De Jong et al, 2022; Tortajada et al, 2012
CFH	196706756	2748C>G	Tyr916*	n/a	2	Pathogenic	De Jong et al, 2022
CFH	196706767	2759G>A	Trp920*	n/a	1	VUS	-
CFH	196709816	2850G>T	Gln950His	0.6	23	Benign	De Jong et al, 2022; Mohlin et al, 2015; Szarvas et al, 2016
CFH	196709833	2867C>T	Thr956Met	0.17	5	Benign	De Jong et al, 2022; Perez-Caballero et al, 2001; Szarvas et al, 2016; Merinero et al, 2018

Supplementary Table 1. Rare Genetic Variants in the CFH and CFI Genes Identified in AMD Patients (continued)

**Gene**	**Genomic position**	**Nucleotide change**	**Protein change [a]**	**Minor allele frequency (%) [b]**	**No. of AMD patients**	**Pathogenicity category rare variant**	**References pathogenicity categories** (functional studies)
CFH	196709874	2908A>G	Ile970Val	0.002	1	VUS	-
CFH	196709875	2909T>C	Ile970Thr	n/a	1	Likely benign	De Jong et al, 2022
CFH	196712608	3160G>A	Val1054Ile	0.003	1	Likely pathogenic	De Jong et al, 2022
CFH	196712674	3226C>G	Gln1076Glu	0.03	1	Likely benign	De Jong et al, 2022; Neumann et al, 2003
CFH	196712682	3234G>T	Arg1078Ser	0.01	2	Likely benign	De Jong et al, 2022
CFH	196716375	3628C>T	Arg1210Cys	0.03	1	Pathogenic	De Jong et al, 2022; Manuelian et al, 2003; Sanchez-Corral et al, 2002; Recalde et al, 2016
CFH	196716399	3652T>G	Cys1218Gly	n/a	1	Likely pathogenic	De Jong et al, 2022
CFI	110662092	1709G>C	Ser570Thr	0.01	1	Benign	De Jong et al, 2020; De Jong et al, 2022
CFI	110662144	1657C>T	Pro553Ser	0.27	14	Likely pathogenic	Bienaime et al, 2010; Bresin et al, 2013; Kavanagh et al, 2015; Geerlings et al, 2017; Java et al, 2019; De Jong et al, 2020; Java et al, 2020
CFI	110662164	1637G>A	Trp546*	n/a	1	Pathogenic	Nilsson et al, 2010; De Jong et al, 2020
CFI	110667387	1420C>T	Arg474*	0.006	2	Pathogenic	Fremeaux-Bacchi et al, 2004; Bienaime et al, 2010; Fakhouri et al, 2010; Nilsson et al, 2010; Kavanagh et al, 2015; De Jong et al, 2020; Java et al, 2020
CFI	110667431	1376A>C	Tyr459Ser	0.0009	1	VUS	Bienaime et al, 2010; Bresin et al, 2013; De Jong et al, 2020
CFI	110667465	1342C>T	Arg448Cys	0.01	5	VUS	De Jong et al, 2020
CFI	110667485	1322A>G	Lys441Arg	0.24	10	Benign	Kavanagh et al, 2015; Hallam et al, 2020; De Jong et al, 2020; Java et al, 2020
CFI	110667590	1217G>A	Arg406His	0.13	4	Benign	Kavanagh et al, 2008; Kavanagh et al, 2015; Java et al, 2019; Hallam et al, 2020; De Jong et al, 2020; Java et al, 2020

Supplementary Table 1. Rare Genetic Variants in the *CFH* and *CFI* Genes Identified in AMD Patients (continued)

Gene	Genomic position	Nucleotide change	Protein change [a]	Minor allele frequency (%) [b]	No. of AMD patients	Pathogenicity category rare variant	References pathogenicity categories (functional studies)
CFI	110670674	1025G>A	Gly342Glu	0.003	1	Pathogenic	De Jong et al, 2020; De Jong et al, *in preparation*
CFI	110670683	1016G>A	Arg339Gln	0.0009	2	Pathogenic	Kavanagh et al, 2015; Java et al, 2020; De Jong et al, 2022
CFI	110678925	898G>A	Ala300Thr	0.03	3	Benign	Kavanagh et al, 2015; Java et al, 2020
CFI	110681474	835A>C	Asn279His	n/a	1	VUS	-
CFI	110681491	818C>G	Pro273Arg	n/a	1	VUS	-
CFI	110681527	782G>A	Gly261Asp	0.19	11	Benign	Nilsson et al, 2006; Servais et al, 2007; Bienaime et al, 2010; Kavanagh et al, 2015; Hallam et al, 2020; De Jong et al, 2020; Java et al, 2020
CFI	110681679	772G>A [f]	Ala258Thr	0.02	3	Pathogenic	Vyse et al, 1996; Ponce-Castro et al, 2008; Kavanagh et al, 2015; Szarvas et al, 2016; Hallam et al, 2020; De Jong et al, 2020; Java et al, 2020
CFI	110682768	563G>C	Gly188Ala	n/a	2	Pathogenic	Van de Ven et al, 2013; De Jong et al, 2020
CFI	110685783	392T>G	Leu131Arg	0.002	5	Pathogenic	Geerlings et al, 2017; De Jong et al, 2020
CFI	110685820	355G>A	Gly119Arg	0.09	20	Pathogenic	Van de Ven et al, 2013; Kavanagh et al, 2015; Geerlings et al, 2017; Hallam et al, 2020; De Jong et al, 2020; Java et al, 2020
CFI	110685847	329-1G>A	-	n/a	1	VUS	De Jong et al, 2020
CFI	110687788	250A>G	Thr84Ala	n/a	2	VUS	De Jong et al, 2020
CFI	110687823	215C>G	Thr72Ser	0.005	1	VUS	De Jong et al, 2020
CFI	110687847	191C>T	Pro64Leu	0.002	2	Pathogenic	De Jong et al, 2020; Kavanagh et al, 2015; Java et al, 2020
CFI	110687890	148C>G	Pro50Ala	0.01	3	Pathogenic	De Jong et al, 2020; De Jong et al, 2022
CFI	110723091	37T>G	Phe13Val	0.002	2	Benign	De Jong et al, 2020
CFI	110723127	1A>G	Lys2_Met138del	n/a	1	Pathogenic [e]	-

Supplementary Table 1. Rare Genetic Variants in the *CFH* and *CFI* Genes Identified in AMD Patients (continued)

Gene	Genomic position	Nucleotide change	Protein change [a]	Minor allele frequency (%) [b]	No. of AMD patients	Pathogenicity category rare variant	References pathogenicity categories (functional studies)
**Carriers of multiple rare *CFH* and/or *CFI* variants**							
CFH<br>*CFI*	196642221<br>110662144	172T>G<br>1657C>T	Ser58Ala<br>Pro553Ser	0.03<br>0.27	1	VUS<br>Likely pathogenic	Merinero et al, 2018; De Jong et al, 2022; Bienaime et al, 2010; Bresin et al, 2013; Kavanagh et al, 2015; Geerlings et al, 2017; Java et al, 2019; De Jong et al, 2020; Java et al, 2020
CFH<br>*CFH*	196711067<br>196706677	3019G>T<br>2669G>T	Val1007Leu<br>Ser890Ile	0.15<br>0.14	2	Benign<br>Benign	De Jong et al, 2022; Tortajada et al, 2012
CFH<br>*CFH*	196646659<br>196709816	481G>T<br>2850G>T	Ala161Ser<br>Gln950His	0.006<br>0.60	1	Likely benign<br>Benign	Sellier-Leclerc et al, 2007; Fakhouri et al, 2010; Servais et al, 2012; De Jong et al, 2022; Mohlin et al, 2015; Szarvas et al, 2016
CFH<br>*CFH*	196646756<br>196654311	578C>T<br>908G>A	Ser193Leu<br>Arg303Gln	n/a<br>n/a	6	VUS<br>VUS	Geerlings et al, 2017; De Jong et al, 2022
CFH<br>*CFH*<br>*CFH*<br>*CFH*	196711067<br>196706677<br>196711098<br>196712624	3019G>T<br>2669G>T<br>3050C>T<br>3176T>C	Val1007Leu [c]<br>Ser890Ile<br>Thr1017Ile<br>Ile1059Thr	0.15<br>0.14<br>0.0008<br>0.03	1	Benign<br>Benign<br>VUS<br>VUS	De Jong et al, 2022; Tortajada et al, 2012
CFH<br>*CFH*	196682946<br>196709816	1418C>T<br>2850G>T	Ala473Val<br>Gln950His	0.01<br>0.60	1	Benign<br>Benign	De Jong et al, 2022; Tortajada et al, 2012; Mohlin et al, 2015; Szarvas et al, 2016
CFI<br>*CFI*	110682768<br>110681527	563G>C<br>782G>A	Gly188Ala<br>Gly261Asp	n/a<br>0.19	1	Pathogenic<br>Benign	Van de Ven et al, 2013; De Jong et al, 2020; Nilsson et al, 2006; Servais et al, 2007; Bienaime et al, 2010; Kavanagh et al, 2015; Hallam et al, 2020; Java et al, 2020
**Total**					**234**		

Rare heterozygous genetic variants in the *CFH* and *CFI* genes, identified in patients with AMD. [a] Based on NM_000186.4, on GRCh37 (hg19) for rare variants in the *CFH* gene, and based on NM_000204.4, on GRCh37 (hg19) for rare variants in the *CFI* gene. [b] Based on the non-Finnish Europeans, as reported in the Genome Aggregation Database (gnomAD) v2.1.1. [c] Homozygous rare variant carrier. [d] based on criteria of the American College of Medical Genetics and Genomics, reporting very strong evidence or pathogenicity frameshift variants in genes where loss of function is a known disease mechanism. [e] This variant leads to loss of the start codon. [f] This variant leads to skipping of exon 5, annotation c.772G>A or r.658_773del; AMD = age-related macular degeneration; *CFH* = complement factor H; *CFI* = complement factor I; VUS = variant of uncertain significance; n/a = minor allele frequency data is not available in the non-Finnish Europeans in gnomAD.
