# Supplementary material for: Geographic Atrophy in Patients with Age-Related Macular Degeneration Is Associated with Rare Variants in Complement Factor H and Complement Factor I
Source: Ophthalmol Sci. 2026 Mar 27;6(6):101171. doi: 10.1016/j.xops.2026.101171 (PMC13218239; doi:10.1016/j.xops.2026.101171)
Supplement: Table S5 [file mmc3.docx]

**Supplementary Table 5.** Age Differences Between Carriers and Noncarriers

| **Category** | **Noncarriers, mean (SD)** | ***CFH* and *CFI* carriers combined, mean (SD)** | ***P* Value** | ***CFH* carriers only, mean (SD)** | ***P* Value** | ***CFI* carriers only, mean (SD)** | ***P* Value** |
| --- | --- | --- | --- | --- | --- | --- | --- |
| All variants | 70.8 (12.4) | 70.6 (12.5) | 0.88 | 68.0 (12.8) | 0.004 | 74.3 (11.3) | 0.001 |
| Pathogenic variants only | 70.8 (12.4) | 68.9 (12.6) | 0.10 | 64.3 (11.0) | < 0.001 | 72.4 (12.7) | 0.28 |

Age differences within the different sub-analyses between the carrier groups and noncarriers, analyzed with independent samples T-tests. CFH = complement factor H; CFI = complement factor I.
