# Supplementary material for: Geographic Atrophy in Patients with Age-Related Macular Degeneration Is Associated with Rare Variants in Complement Factor H and Complement Factor I
Source: Ophthalmol Sci. 2026 Mar 27;6(6):101171. doi: 10.1016/j.xops.2026.101171 (PMC13218239; doi:10.1016/j.xops.2026.101171)
Supplement: Table S6 [file mmc4.docx]

**Supplementary Table 6.** Genetic Risk Scores Between Carriers and Noncarriers

| **Category** | **Sub-analysis** | **Noncarriers, mean (SD)** | ***CFH* and *CFI* carriers combined, mean (SD)** | ***P* Value** | ***CFH* carriers only, mean (SD)** | ***P* Value** | ***CFI* carriers only, mean (SD)** | ***P* Value** |
| --- | --- | --- | --- | --- | --- | --- | --- | --- |
| All variants | GRS based on all 52 AMD-associated variants | 1.38 (1.24) | 1.45 (1.35) | 0.59 | 1.22 (1.33) | 0.25 | 1.74 (1.32) | 0.02 |
|  | GRS based on common AMD-associated variants only (=45/52) | 1.41 (1.21) | 1.27 (1.26) | 0.26 | 1.16 (1.25) | 0.08 | 1.41 (1.27) | > 0.99 |
| Pathogenic variants only | GRS based on all 52 AMD-associated variants | 1.38 (1.24) | 1.69 (1.57) | 0.16 | 1.01 (1.51) | 0.18 | 2.10 (1.48) | 0.002 |
|  | GRS based on common AMD-associated variants only (=45/52) | 1.41 (1.21) | 1.13 (1.32) | 0.13 | 0.83 (1.01) | 0.03 | 1.32 (1.47) | 0.70 |

Genetic risk scores (GRS) within the different sub-analyses. GRSs between the different carrier groups were compared with the noncarriers with independent samples t-tests. CFH = complement factor H; CFI = complement factor I; SD = standard deviation; GRS = genetic risk score.
